# Event and Comment

**Published:** 1905-09-15

**Authors:** 


THE
DENTAL REGISTER.

Vol. LIX. September 15, 1905. No. 9.

### EVENT AND COMMENT.

### The National Dental Association Meeting.

The meeting was held in Buffalo, July 25th to 28th. The section meetings were held in the Iroquois Hotel, the general sessions in the Y. M. C. A. Building and the clinics in the dental college. There was a good attendance of probably not over 600. This is a fairly good average attendance, and is all that could be expected of a body whose membership does not exceed 500. One of the puzzling questions is, why more dentists do n not belong to this society? Some say because the politicians run it; others because its programs are not attractive to the average practitioner; others that the section method of working is a back number, and still others think it costs too much to attend its meetings which require long travels and high expenses at the resort places where it generally meets. It is very evident that the six thousand dentists whom the president said should belong and attend its meeting, do not, and there is no prospect that they are likely to do so in the near future, as the objections noted above will always be valid ones, for there is no practicable way of changing them. Would it not be a good idea to let things follow the natural course and give up all, thought of a national association which shall convene at a given place at some special time? Why not district the country and have a section of the association meet in each district every year and hold conventions or meetings of such character as may seem best fitted to reach the masses of that particular district. These sections could also meet at seasonable times of the year. In connection with this idea we should have a central national meeting each year, made up of delegates from these sections. The delegates should be selected only from those who have presented to their particular sections contributions of such technical or scientific value as should be presented for discussion by similar delegates from all the other sections. Each delegate's expenses being defrayed by the section which sends him. Some such plan would stimulate work in all the sections and the best minds would come together once a year and consider every step of progress made in the whole country. These sections which may be state or district organizations, would have the benefit in the printed transactions of the best work done in all the country. In this way not only would research be stimulated, but politics would not rule or worry anybody, the programs would be of the best character, and the expense of traveling long distances by the many would be obviated. This plan would overturn the present methods by a kind of revolution, but it may be necessary to adopt radical measures to make justifiable progress.

At the recent meeting many good papers were read, but there were so many papers and these were being read simultaneously in three different rooms of the hotel, so that it was next to impossible to secure a proper consideration of any paper. In our judgment it is this very thing that kills the interest of writers in our national meeting.

The clinics were numerous and very well managed. There were good facilities and a chance for everyone to see the demonstrations. The chairman of clinics deserves much praise for his business-like method of securing and presenting the modern technical methods. A large clinic is a difficult problem and can only be successfully managed when an idea is behind the whole transaction, and there is a man of executive force to carry it out.

The meeting goes to Atlanta, Georgia, next year, and to secure more favorable weather, the time has been set for the third Tuesday in September, which will prevent most college men from attending, as it comes so near the time of the opening of the schools. This may be a good thing too, as it will give the folks who are not accustomed to talking, a chance to break in.

The officers for next year are: Dr. M. F. Finley, of Washington, President; Dr. Frank Holland, of Atlanta, Vice-President for the South; Dr. Wm. Conrad, of St. Louis, Vice President for the West; Dr. L. P. Bethel, of Columbus, Ohio, Vice President for the East; Dr. A. H. Peck, of Chicago, Secretary; Dr. V. H. Turner, of Raleigh, N. C., Treasurer.

There was the usual contest over the printing of the proceedings, the *Digest*, being "out-bid" by the *Cosmos*, lost the transactions for the ensuing year. If the expenses of publication are as much as the former publishers declare, it is hard for the spectator to appreciate the eagerness with which certain publishers "cough up" the required funds. The proposition of an Association Journal was again brought up and a committee appointed to report next year on the feasibility of the proposition. We don't see how any committee can report favorably with so small a supporting membership as the society now has.

**The National Association of Examiners.**

This body held its annual meeting the first of the week beginning on Monday, and had concluded its work before the faculty association convened, which was on Thursday. This was a source of considerable annoyance to some members of the Faculties Association, as it prevented a conference of the two organizations as regards the standards of reputability which the examiners have adopted. There was a large meeting of the examiners and some important business was transacted. The most important was the re-affirming of the standard of college education as adopted last year, and the publication of a list of twenty-one out of fifty-three colleges that had already qualified under the new standard. The publication of this list by one of the city newspapers made opportunity for some warm discussions in the hotel corridors, as the gentlemen connected with schools which had not as yet assented to the new standard thought they had been blacklisted. This was, however, made clear when it was explained that no school had been blacklisted and could not be until the time set for the new rules taking effect, and even then such schools as do not agree to adopt the standard will do it from choice and not by coercion. It may be that there is to be a contest between the colleges and the examiners, but at present it looks as though there would be a peaceful adjustment. Another matter which this body has undertaken is to ascertain the reputability of colleges by examining their graduate product. The plan is to have every state examining board report the result of their examinations to the National Association of Examiners. These reports will include complete statistics as to the results of the examination of students from all the colleges. Any college whose graduates fail to the extent of 50 percent shall not be considered a reputable school, and until its methods of teaching have been improved, its graduates shall not be eligible for examination. This is a truly scientific method of determining a question of this kind, and it will be interesting to note results. It looks as though the examiners were either going into trouble or are to get some other people into trouble. If, after all, these or any other measures will place our professional education on a proper and a uniform basis in all the states, we shall soon have a national standard and basis, for which we shall all be grateful, and be satisfied with any measures that shall accomplish this much desired result.

The officers for next year are: President, H. W. Campbell, of Suffolk, Va.; Vice Presidents, F, O. Hetrick, Ottawa Ka., F. A. Shotwell, Rogersville, Tenn., G. E. Mitchell, Haverhill, Mass.; Secretary and Treasurer, C. A. Meeker, Newark, N. J.

### National Association of Dental Faculties.

The meeting of this body was begun Thursday afternoon and continued until Friday evening. The president, Dr. S. W. Foster, of Atlanta, Ga., was too ill to attend, and Dr. J. D. Patterson presided. Several prominent members of this body were absent, and not much business of a puplic character was considered. The resolution offered last year in regard to raising the entrance requirements to a high school graduation in 1906, was brought up and laid on the table indefinitely without discussion; just why we were unable to learn. Possibly because the examiners had taken this matter, as well as the length of the term into their hands for adjustment, it was thought best to let the matter work out that way. It was expected that there would be a considerable contest over this matter. By a good majority, the colleges voted to raise their fees next year to one hundred and fifty dollars. This has become almost necessary, because of the great increase in the cost of maintaining facilities for up-to-date instruction. Many of the schools have already adopted the increase fees. This action may cause some schools to go out of business and may lessen the number of students who will go into the profession, but the schools that exist will certainly be better off in revenue even should there be some loss in students. There seemed to be a feeling that progress of a substantial character was being made in educational affairs, and the character of the discussions on business matters indicated a better understanding of what the duty of the college was to be toward the profession.

Consul Worman was present at the last session of the association, and made an address on the subject of the bogus dental degree, which he has worked so hard to have driven out of Germany. He made a very encouraging report on the present conditions, as through his work the whole German Empire had been instructed as to the difference between the bogus and genuine degree. He made a most earnest plea for the most careful surveilance in this country, and the revoking of all charters for dental colleges, which were not known to be unqualifiedly reputable. He also pleaded for a uniform standard of education and the unification of methods of education in all schools, so that foreign countries could know without question that our degrees mean certain standards. He advised state supervision and inspection of colleges, so that their reports might be available to the foreign consulates in this country, as they are frequently called upon to report on such matters. He believes with a united profession under a single adequate standard that the American D. D. S. degree will be protected abroad as well as at home. The officers elected for next year are: President, Dr. J. H. Kennerly, of St. Louis; Vice President, Dr. John Hart, of New York; Secretary, Dr. G. E. Hunt, of Indianapolis; Treasurer, Dr. H. R. Jewett, of Atlanta.

